# Clinical and radiological effects of Bevacizumab for the treatment of radionecrosis after stereotactic brain radiotherapy

**DOI:** 10.1186/s12885-024-12643-6

**Published:** 2024-07-30

**Authors:** Teuta Zoto Mustafayev, Menekse Turna, Yasemin Bolukbasi, Evrim Tezcanli, Yildiz Guney, Fazilet Oner Dincbas, Beste Melek Atasoy, Gamze Ugurluer, Hale Basak Caglar, Banu Atalar, Enis Ozyar

**Affiliations:** 1 Acibadem Maslak Hospital, Department of Radiation Oncology, Istanbul, Turkey; 2 Department of Radiation Oncology, Anadolu Medical Center affiliated with Johns Hopkins Medicine, Kocaeli, Turkey; 3 Department of Radiation Oncology, Koc University School of Medicine, Istanbul, Turkey; 4 Department of Radiation Oncology, Acibadem Altunizade Hospital, Istanbul, Turkey; 5 Department of Radiation Oncology, Memorial Ankara Hospital, Ankara, Turkey; 6 Cerrahpasa Medical School, Department of Radiation Oncology, Istanbul University-Cerrahpasa, Istanbul, Turkey; 7 Department of Radiation Oncology, Memorial Bahcelievler Hospital, Istanbul, Turkey; 8 Department of Radiation Oncology, Marmara University School of Medicine, Istanbul, Turkey; 9 Department of Radiation Oncology, Acibadem MAA University School of Medicine, Istanbul, Turkey

**Keywords:** Radiation induced toxicity, Brain metastasis, Stereotactic radiosurgery, Bevacizumab

## Abstract

**Purpose:**

The purpose of this multicenter retrospective study was to analyze the clinical and radiological effects of bevacizumab (BV) on radionecrosis (RN) that developed after stereotactic radiotherapy (SRT) for brain metastasis.

**Methods:**

Forty patients with SRT related symptomatic brain RN treated in 10 radiation oncology centers were analyzed. The clinical response to BV treatment was categorized as follows: complete (no additional treatment required), partial (requiring either steroids or repeat BV), and unresponsive (requiring surgery). The radiological features of brain RN were analyzed in 10 patients whose serial MRI scans were available after corticosteroid and BV treatments.

**Results:**

BV was used as a first line treatment in 11 (27.5%) and as a second line treatment in 29 (72.5%) of patients. The neurological symptoms regressed in 77.5% of patients after treatment with BV (45% complete response, 32.5% partial response). The median edema volume increased from 75.9 cc (range: 5.9-125.8 cc) at RN to 113.65 cc (range: 1.5-382.1 cc) after use of corticosteroids, representing a rate of 39.8% increase (*p* = 0.074). However, after BV treatment the median volume of edema decreased to 19.5 cc (range: 0-163.3 cc) which represents a difference of 62.2% (*p* = 0.041) from RN.

**Conclusion:**

The use of BV caused clinical response rate of 77.5% and a good radiological response in corticosteroid unresponsive patients. The role of BV should be further investigated in prospective studies.

**Supplementary Information:**

The online version contains supplementary material available at 10.1186/s12885-024-12643-6.

## Introduction

The increased use of ablative dose in the brain with stereotactic radiosurgery (SRS) or stereotactic radiotherapy (SRT) is leading to a high frequency of symptomatic and asymptomatic radiation necrosis (RN), ranging from 2.5% up to 24%, depending on different radiological definitions of RN (whether only morphological features of MRI are used, or more advanced calculations based on data from spectroscopy and perfusion are added) [1]. Various methods have been used for the treatment of symptomatic RN, with varying degrees of success; including surgery, high dose steroids, hyperbaric oxygen, laser interstitial thermal therapy, heparin, warfarin, pentoxifylline, and vitamin E [2].

Previously, Bevacizumab (BV), an anti-VEGF antineoplastic drug, used in the treatment of metastatic colorectal cancer and recurrent primary brain tumors, was reported to be effective in the treatment of RN by decreasing tumor vascularization and vascular permeability which leads to decrease in perfusion and edema, the main culprits of RN development [3]. After the pioneering proof of concept study [4], BV has been studied in few prospective studies which included patient with RN after irradiation to primary brain or head and neck tumors, showing promising results [5–7]. In some of these studies a decrease in both T1 and T2/FLAIR volumes accompanied the clinical response. The efficacy of lower dose BV was also studied in another prospective trial achieving symptomatic relief in 90% of patients [8]. As mentioned, most of the earlier prospective trials [5–7] included patients with RN from primary brain or head and neck tumors and only one study included exclusively BM patients [8]. Thus, the good response to BV on RN following BM treatment was extrapolated from these earlier limited prospective trials. Despite few trials showing the durability of BV’s effect and its safety, most retrospective trials including patients who developed RN after SRT for BM have shown promising results [9, 10]. Although the number of patients and studies is limited, the achievement of a good response accompanied by a predictable and avoidable toxicity profile have made BV one of the two main medical treatment of RN, alongside the surgical approach which is mostly used in emergency or refractory cases [2]. However, from the current literature it is unclear which factors can contribute to better response to BV specifically in patients developing RN after SRT for brain metastasis. Furthermore, the radiological and clinical effect of BV in patients with refractory symptoms after high dose dexamethasone is not studied.

While the results of further prospective trials that would shed light into the effect of BV are eagerly awaited [11], in this study we retrospectively analyzed the clinical and radiological effects of BV and dexamethasone in patients developing RN after SRT for brain metastasis, which factors contribute to the difference in response to BV and to what extent the change in radiological findings reflect the clinical outcome.

## Methods

### Patients

Forty patients who were treated from November 2011 to July 2020 with at least one course of SRS/SRT due to BM and subsequently developed symptomatic RN treated with BV +/- corticosteroids were included in this retrospective multicenter study. Patients’ data were collected from 10 centers in Türkiye [Acibadem Adana Hospital (1) Acibadem Altunizade Hospital (1), American Hospital (1), Marmara University Hospital (1), Memorial Bahcelievler Hospital (1), Acibadem Kozyatagi Hospital (3), Memorial Ankara Hospital (3), Koc University Hospital (4), Acibadem Maslak Hospital (12), Anadolu Medical Center (13). Patients treated with Cyberknife, Gamma knife or Linac based systems were included in the study. Inclusion criteria were: (1) development of radiological signs consistent with RN after brain SRS/SRT, (2) grade 2 or higher neurological deficits or grade 3 or higher increased intracranial pressure symptoms – ICP (i.e., headache, nausea, vomiting) according to Common Terminology Criteria for Adverse Events (CTCEA) version 4, in the presence of radiological signs of RN, (3) receiving at least one cycle of BV, (4) a minimum follow-up of at least two months after starting of the BV treatment. Biopsy was not a requirement for RN diagnosis. This retrospective study was approved by the local ethical committee (N: 2022-04/06; Date: 25.02.2022).

### Radiological diagnosis

Radiological diagnosis of RN was given by the radiologists of each participating center based on multiparametric MRI images, including perfusion, diffusion, and spectroscopy. Radiologically, RN was defined as lesions showing increase in lactate peak and decrease of the other metabolites in spectroscopy, decrease in perfusion (low relative cerebral blood volume (rCBV)), and centrally restricted diffusion as suggested by the apparent diffusion coefficient (ADC) mapping [12]. Presence of large edema and mass effect in T2/FLAIR, “soap-bubble” or “cut green pepper” enhancement in T1 gadolinium images were complementary for the diagnosis [13].

All patients had to have a documented multiparametric MRI at diagnosis of RN. In addition, if patients had other treatments for RN before BV, MRI images after that treatment but before BV were analyzed as well. The minimum requirements for follow-up MRI were the availability of T2/FLAIR and T1 contrast enhanced sequences. Additionally, a response MRI within 1–2 months after the BV treatment was required in surviving patients.

### Treatment assessment

*Clinical assessment (all patients)*:

1. **General clinical response**: The response to BV treatment was classified by the treating physician as complete (not requiring additional treatment), partial (needing steroids or repeat BV), and unresponsive (requiring surgery), within one month after treatment discontinuation.
2. **Neurological deficit response**: Presence and type of neurological deficits was recorded at the RN diagnosis and at the first follow up after BV use.

#### Radiological assessment

In addition to the mandatory MRI at RN, 10 out of 28 patients who were given BV as second line treatment after high dose corticosteroid use, had available MRI after corticosteroid as well as after BV treatment. For each MRI, the volumes of edema (in T2/FLAIR sequence) and contrast-enhanced lesions (in T1 contrast sequence) were determined separately by contouring the lesions at the time of RN, after corticosteroid use, and after BV. In order to decrease physician dependent variation, contouring was done by one physician (TZM) using available treatment planning system workstation [Eclipse treatment planning system (TPS) (Varian Medical Systems, Palo Alto, CA)]. T1 contrast enhanced, and T2/FLAIR volume measurements were preferred over bidirectional diameter measurements because they were reported as more reliable according to the prospective study by Levin et al. [5]. The intensity of contrast enhancement decrease was not measured. Even in cases when lesions had a noticeable lower contrast uptake after BV use, the T1 lesion measurement included the lesion cavity and the remnant lesion, even though the uptake was minimal.

#### Follow up

Patients were followed-up and assessed for neurological symptoms at every BV session during the treatment and every 2–3 months afterwards.

### Statistical analysis

The Wilcoxon signed rank test was used to compare the change in T2/FLAIR, and T1-weighted gadolinium enhancement volume at RN, after steroid use and after BV timepoints. Risk ratio was calculated to estimate the effect of factors that might affect the response to BV such as age, gender, presence of comorbidity, presence of neurological deficit, metastasis diameter, primary tumor type, BV dose, BV cycle number, timing of BV and whether BV was the first or the second-line treatment. Fisher’s exact test was used in cases when risk ratio analysis was not feasible. The statistical analyzes were done with SPPS (IBM SPSS Statistics for Windows, version 23, IBM Corp., Armonk, N.Y., USA). Assessments were two sided and *p* < 0.05 was considered as statistically significant.

## Results

Patients and lesions characteristics are summarized in Table 1. More than half of the patients (57.6%) had received either whole brain radiation therapy (WBRT) or, re-SRT. The median BED_3_ dose of SRS/SRT was 108 Gy (Range: 51.3–216 Gy), while the total median BED_3_ dose at BM lesions who developed RN was 148 Gy (range: 51.3–346 Gy). Beside the RN lesions, 28 patients (70%) had other synchronous or metachronous BM lesions treated with SRT. Twenty patients (50%) were treated with Cyberknife, 12 patients (30%) with Linac based platforms and 8 patients (20%) with Gamma knife.

**Table 1:** Patients and lesions characteristics

Characteristic	Number (%)
Patient number	40 (100%)
Age (median, range)	55 years (38–79 years)
Sex Male Female	24 (60%) 16 (40%)
Comorbidity Diabetes *CVD	5 (12.5%) 8 (20%)
Primary diagnosis Lung Breast Renal cell carcinoma Other	24 (60%) 10 (25%) 2 (5%) 4 (10%)
Metastasis diameter (mm, median, range)	19.5 mm (2–45 mm)
Metastasis volume (cc, median, range)	2.3 cc (0.01–32.1 cc)
Lesion location Frontal Parietal Temporal Occipital Brain stem Basal ganglia Other	12 (30%) 14 (35%) 6 (15%) 1 (2.5%) 2 (5%) 2 (5%) 3 (7.5%)
RT treatments on RN lesion before SRS **WBRT Previous SRS WBRT + previous SRS None	13 (32.5%) 6 (15%) 4 (10%) 17 (42.5%)
Other brain lesion SRT	28 (70%)
SRS/SRT total dose (median, range)	20 Gy (11–24 Gy) / 27 Gy (21–30 Gy)
SRT fraction number (median, range)	3 (3-5)
†BED_3_ of SRS/SRT (median, range)	153 Gy (51.3–216 Gy) / 99 Gy (67–108)
‡BED_10_ of SRS/SRT (median, range)	60 Gy (23.1–82 Gy) / 49.6 Gy (35.7–51.3)
Total BED_3_ (median, range)	148 Gy (51.3–346 Gy)

*CVD-cardiovascular disease.

**WBRT-Whole Brain Radiation Therapy.

†BED_3_-Biologically Effective Dose for late responding tissue, alpha/beta equals 3.

‡ BED_3_-Biologically Effective Dose for early responding tissue, alpha/beta equals 10 BED = nd[1 + d/(α/β)] where n = number of fractions, d = fraction dose.

**Fig. 1:**
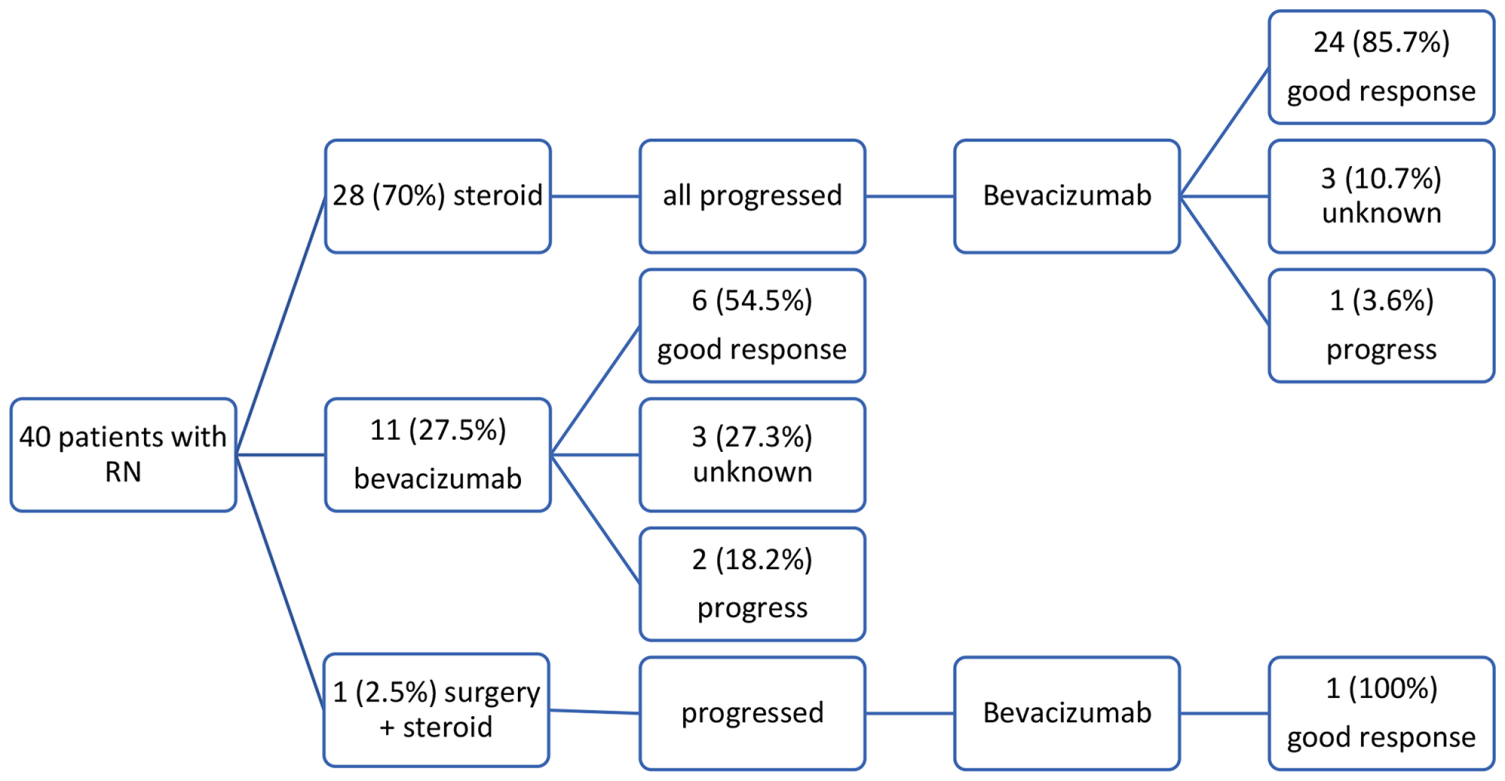
Scheme of clinical response according to initial treatments

RN developed at a median of 11.5 months (range 2–39 months) after SRT. At the time of RN diagnosis 65% of patients had KPS 90 and higher. While the majority patients (62.5%) suffered only increased intracranial pressure related symptoms such as headache, nausea, vomiting, in 15 patients (37.5%) other neurological deficit and symptoms including sensory-motor deficit, speech and balance impairment and seizures were present (Table 2). First treatment choice was corticosteroids in 28 patients (70%), BV in 11 (27.5%) patients, and both corticosteroids and surgery in one (2.5%) of patient. Median number of BV cycles was 4, and 92% of patients had 2–8 cycles, with half of them receiving doses of 4 and 6. One patient had 1 cycle and 2 patients had more than 8 cycles. Six out of 11 patients who received BV as first line treatment did not receive further treatment, three had unknown response, and the remaining two patients underwent surgery due to persistence of the symptoms (Fig. 1).

**Table 2:** Patient and treatment characteristics

Characteristic	Number (%)
**Time from last SRT to RN (median**,** range)**	11.5 months (2–39 months)
**KPS** **≥** **90** **<90**	26 (65%) 14 (%35)
**Neurological deficit**	15 (37.5%)
**Neurological deficit type** **Sensory deficit** **Motor deficit** **Speech difficulty** **Imbalance** **Seizures**	5 (12.5%) 3 (7.5%) 4 (10%) 2 (5%) 1 (2.5%)
**First line treatment** **Corticosteroids** **Corticosteroids + surgery** **BV**	28 (70%) 1 (2.5%) 11 (27.5%)
**Second line treatment** **BV** **Surgery** **No further treatment**	29 (72.5%) 2 (5%) 9 (22.5%)
**Time from RN diagnosis to BV initiation**	1 month (0–13 months)
**BV cycle number (median**,** range)**	4 (1–26)
**BV dose** **5 mg/m2** **7.5 mg/m2** **Other**	8 (20%) 15 (37.5%) 17 (42.5%)

### Clinical response analysis

A good clinical response (complete or partial) was noted in 31 (77.5%) of patients, while progressive disease was observed in 3 (7.5%) patients when assessed 1 month after completion of the BV treatment. Clinical objective response could not be assessed in 6 (15%) patients; 3 patients died before the completion of the planned treatment and 3 patients had stable disease during the treatments but no other follow up visits. These 6 patients were considered as not having a good response. A schematic representation of clinical response according to the initial treatment is shown in Fig. 1.

No correlation was found between response and sex, presence of comorbidity, presence of neurological deficit, metastasis diameter, primary tumor type, BV dose, BV cycle number, timing of BV and whether BV was the first or the second-line treatment (Supplement Table 1). It was observed that all unresponsive patients were older than 55 years old.

Age older than 55 years was the only factor associated with a bad response to BV (Fisher’s exact test *p* < 0.0001).

The neurological deficit in 15 patients at presentation disappeared in 9 patients (60%) and persisted in 6 (40%). None of the factors analyzed influenced the presence of neurological deficit after the treatment.

### Follow up

During a median follow up of 10.5 months (2–62 months), neurological deficit occurred de novo in 8 patients. In the last follow up, a total of 14 (35%) patients had either persistent or newly developed neurological deficits.

### Radiological response analysis

**Fig. 2:**
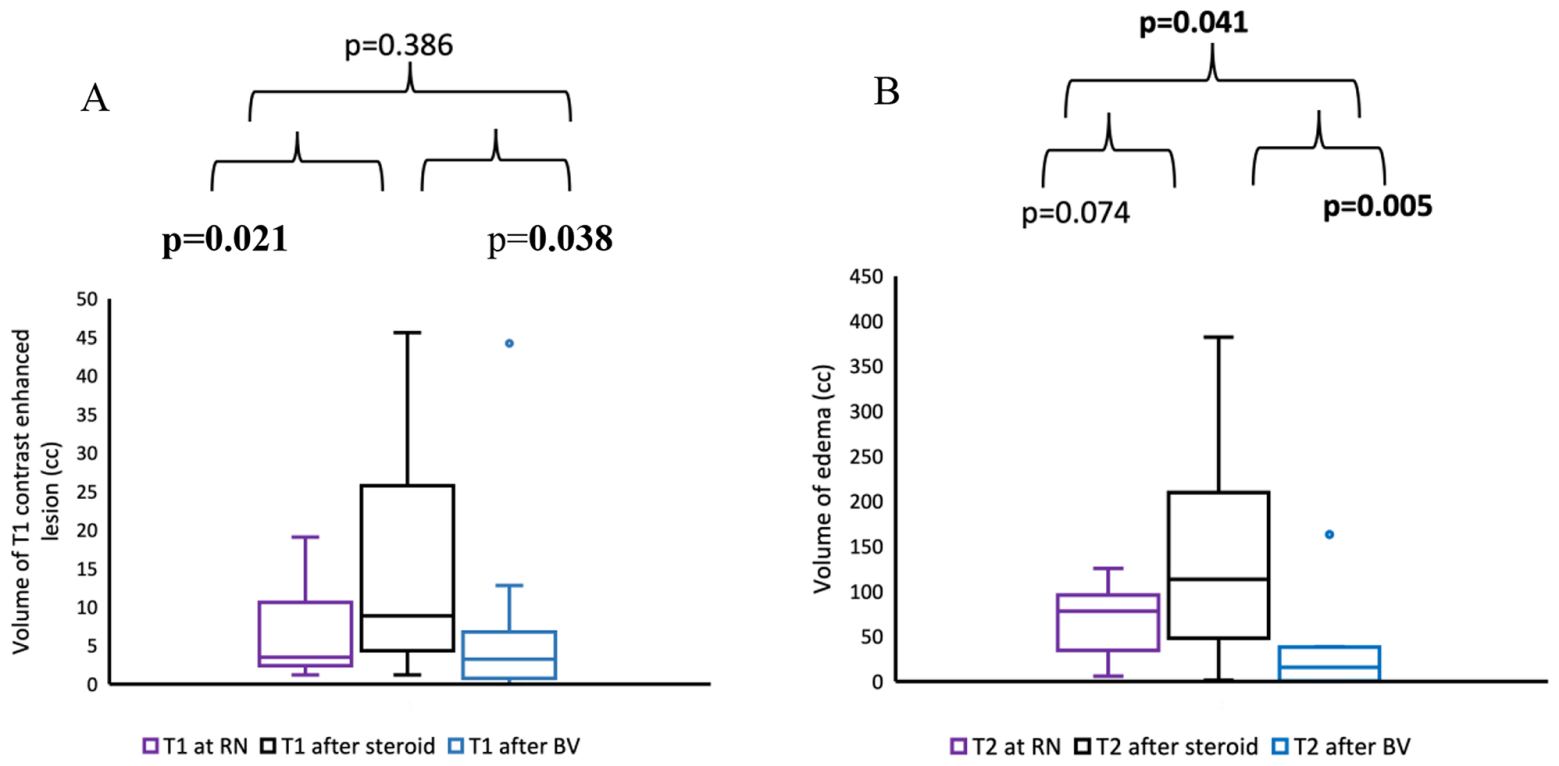
Change in (**A**) T1 contrast enhanced lesion volume and (**B**) T2 edema volume at RN diagnosis, after corticosteroids use and after BV treatment

**Fig. 3:**
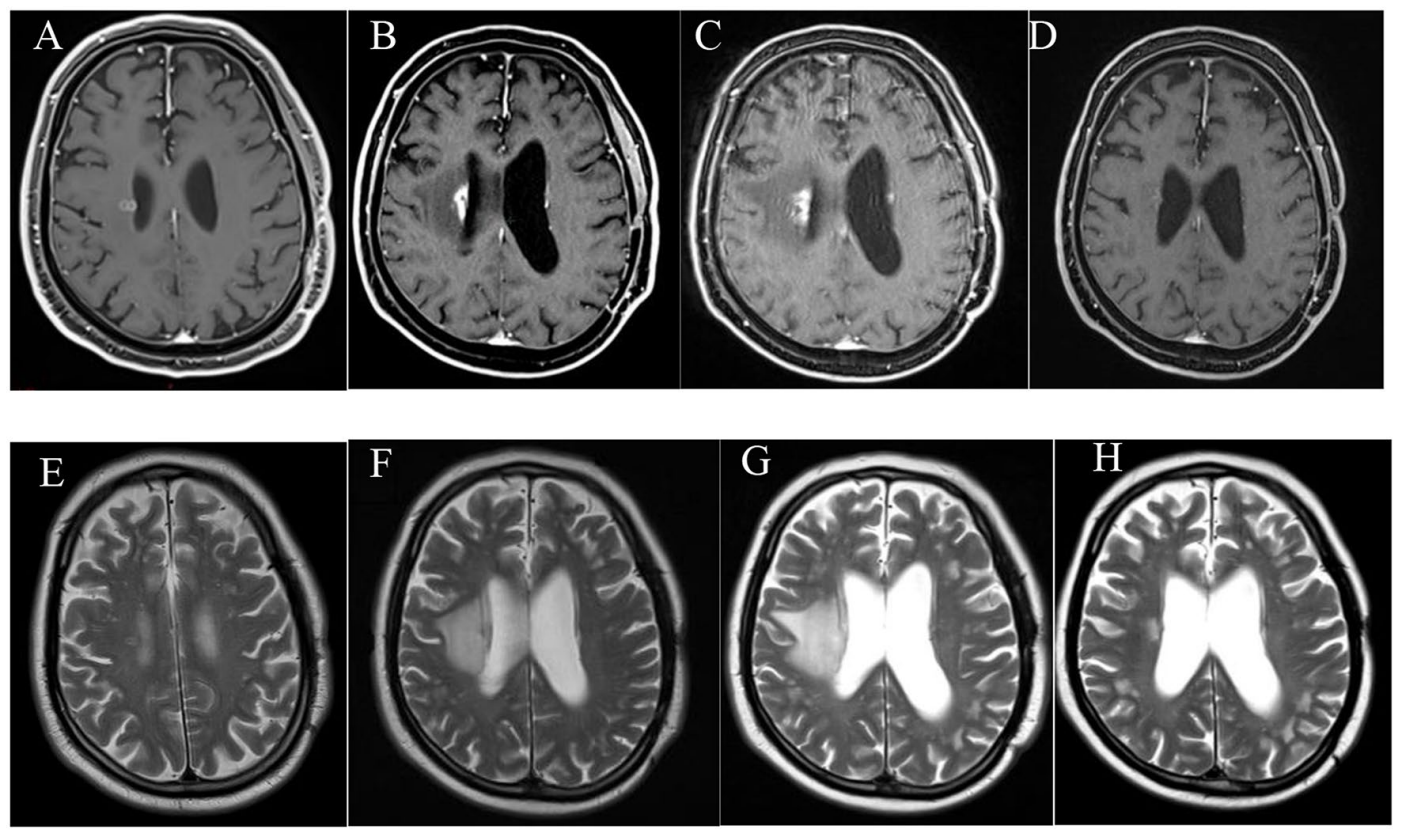
MRI images of a lung cancer patient with BM developing RN after SRS and treated with corticosteroids and BV. (**A**) Metastatic lesion adjacent to right lateral ventricle, (**B**) T1 weighted contrast enhanced lesion at the time of RN diagnosis. (**C**) T1 weighted contrast enhanced lesion after 1 month, after use of high dose dexamethasone. (**D**) T1 weighted contrast enhanced lesion after 3 months, after use of BV. (**E**) T2 weighted image showing metastatic lesion adjacent to right lateral ventricle. (**F** T2 weighted image showing edema at the time of RN diagnosis. (**G**) T2 weighted image showing edema after use of high dose dexamethasone. (**H**) T2 weighted image showing edema after use of BV

Ten out of the 28 patients which were treated with corticosteroids before BV use, had available radiological data for retrospective evaluation with MRI at RN diagnosis, after corticosteroid treatment and after BV treatment. The radiological analysis was performed only for these 10 patients. The change in the volume of T1 weighted contrast enhanced lesion and T2 weighted edema at the three MRI images are shown in Fig. 2. There was a non-significant 25% decrease of T1 contrast enhanced lesion volume in MRI images (*p* = 0.386) despite a 62.2% statistically significant decrease in T2 edema volume (*p* = 0.041) from RN diagnosis to after BV treatment. One example of treatment response is shown in the MRI images in Fig. 3. All these patients had either a complete or partial response initially. Five out of 6 patients who had an increased/stable T1 lesion volume at the post BV MRI as compared to the MRI at RN diagnosis had either a persistent or new neurological deficit, while none of 4 patients with decreased T1 lesion volume had persistent or new neurological deficit (Fisher’s exact test *p* = 0.048).

## Discussion

The results of our retrospective study have shown that use of BV resulted in improvement of the clinical symptoms in 77.5% of patients for the treatment of SRT induced symptomatic brain RN and this result is consistent with previous studies which reported symptom improvement rate of 62.1–100% (Table 3). Most of the early studies included patients with RN from radiotherapy to non-metastatic lesions, e.g., primary brain tumors, or head and neck tumors. Similarly, the effect of BV was shown in patients with RN after SRS for brain metastases as well. In a meta-analysis of 89 patients from 12 retrospective studies which included RN patients after BM SRS reported that 88% of patients had either complete resolution or improved symptoms [10]. In one study [15] 40 patients were retrospectively investigated for the radiological and clinical effect of BV on RN after BM treatment and as a result they showed a significant increase in KPS. Similarly in a recent study [16] where 45 patients were included, 15 of which were metastatic, it was shown that BV achieved a response in four-fifth of the patients. There are also retrospective studies that are unable to show the benefit of BV. In one study where 11 patients were included, single BV dose was unable to cause durable response, although a temporary relief was observed in all patients as shown by the decrease or discontinuations of corticosteroids [18]. In another study where 15 patients were included, they were unable to show an improvement in performance status despite a reduction in steroid use in five of the patients [19].

**Table 3:** Summary of selected studies of BV treatment after brain radiation necrosis (omitted studies with exclusively glioma patients)

Study	Protocol	No of pts	Lesion type	BV dose/ cycles	Clinical response	T1 radiologic response	T2 radiologic response	Serious (grade > 3) side effects	Relapse
[5]	Prospective RCT (with crossover)	14	Brain tm/ H&N	7.5 mg/kg 4 cycles	All treated patients	63% volume decrease	59% volume decrease	2 thrombosis 1 asp pneumonia	2 patients
[6]	Prospective RCT	112 /58 BV	NF ca.	5 mg/kg 4 cycles	62.1% vs. 42.6%	25.5% volume decrease	51.8% volume decrease	1 ischemic stroke	12/58 on BV arm
[8]	Prospective, single arm	21	Metastasis	1 mg/kg 3 cycles	90%	95.2% intensity decrease	95% volume decrease	No Grade > 2	Not reported
[14]	Prospective, single arm	10	Mostly AVM	2.5 mg/kg 1 dose	80%	74% volume decrease	50% volume decrease	No serious side effects	2 patients
[10]	Meta-analysis (mostly retrospective)	89	Metastasis	various	88%	47.03% volume decrease	61.78% volume decrease	1/63 of reported Grade 3 pulm. thrombosis	15 patients
[9]	Systemic review (mostly retrospective)	236	Metastasis/ brain tm/ H&N	various	91%	50% volume decrease	59% volume decrease	5 Gr3: 3 thrombosis 1 ischemic stroke 1 asp pneumonia	46/135 reported (34%)
[15]	Retrospective	40	Metastasis	various	67.5%	75% volume decrease	76.2% volume decrease	No serious side effects	Not reported
[16]	Retrospective	45	Metastasis/brain tm/ other	7.5 mg/kg 4 cycles		49.3% volume decrease	62.3% volume decrease	2 cerebral hemorrhages	8 patients
[17]	Retrospective	95 (41 BV)	Metastasis	various	27/41 BV patients (65.9%)	28.7% diameter decrease	43.7% diameter decrease	2 Gr. 4 bleeding 1 Gr. 3 GIS toxicity 1 Gr 3 allergy	23/41 BV patients (56%)
This study	Retrospective	40	Metastasis	various	77.5%	25% volume decrease	62.2% volume decrease	Not reported	8 patients

A new finding of this study is determining older age (i.e. 55 years) as the only clinical predictor for lack of response to BV. In one of the studies that investigated factors affecting response to BV that was done in an heterogenous group (both glioma and non-glioma patients) BV was more effective in non-glioma patients and in patients without diffusion restriction. In another study, where none of the patients had RN due to metastasis treatment, radiological parameters (baseline K^trans^ and post BV K^trans^ value) were predictive of initial BV response and relapse, respectively [20].

Another finding of this study is improvement of most ICP symptoms without durable decrease of neurological deficits. The radiological data of our 10 patients might shed some light on this discrepancy. In the studied cases where BV was given as a second line treatment it was able to decrease edema (T2 weighted) significantly and probably cause the decrease in ICP symptoms and allowed those patients to be classified as having good response. However, there was not a meaningful decrease in T1 weighted contrast enhanced lesion/RN related cavity size after BV and patients with the no decrease or even increase in T1 contrasted lesions had more persistent neurological deficits or newly developed ones. There was a meaningful increase in T1 contrasted lesion volume despite a non-significant minimal increase in T2 weighted edema during corticosteroids use in the corticosteroid refractory patients. Although the number of patients is small to draw a robust conclusion, when we consider the radiological data, we can assume that in corticosteroids refractory patients during corticosteroids use edema remains stable but the contrasted cavitary lesion increases in size, leading to persistent ICP and increase neurological deficits. BV reduces edema and symptoms below the levels at RN diagnosis, but it is ineffective to decrease T1 contrast cavitary lesions at the same extent (i.e. persistent/recurrent neurological deficit). Whether BV could be able to decrease T1 contrast enhanced/cavitary lesions if given immediately at RN diagnosis and not after corticosteroids use is arguable since some of the patients who were given BV at RN diagnoses also had persistent neurological deficit as well. Additionally, timing of BV or whether it was given immediately after RN or after corticosteroids use did not significantly correlate with neither general clinical response nor neurological deficiency. When we compared our radiological data with previous studies, we noticed a comparable decrease in T2 weighted edema volume (62.2% in our study vs. 43.7–95% in other studies, Table 3). However, the reduction in T1 weighted volume was smaller in our study when compared to the other studies (25% in our study vs. 25.5–75% in other studies, Table 3).

Some of the questions that remain unanswered are: which are the criteria to refer the patients immediately to BV without prescribing corticosteroids first, what should be the starting time, schedule, and dose of BV to avoid persistence of neurological deficits even after radiological improvement. Also, what should be the approach to patients that are at higher risk of being unresponsive, such as older patients.

The main limitation of our study is its retrospective nature and short follow-up time (median follow up after BV 10.5 months). Inability to analyze and compare MRI images for all patients is one important limitation. Another handicap of the study is the lack of patient reported clinical response, neurocognitive testing and lack of a conclusive physician reported response in six patients. Also, patients were not analyzed for BV related side effects.

As a conclusion, BV is an effective treatment option for SRT induced RN, it successfully decreases edema and neurological symptoms in most patients, even in patients unresponsive to steroid treatment. The main mechanism might be the significant reduction in edema volume, which improves ICP symptoms. Lack of decrease in T1 weighted contrast enhanced lesion and cavity volume may lead to the persistence or development of neurological deficits. Patients younger than 55 years old benefit the most from BV treatment.

### Electronic supplementary material

Below is the link to the electronic supplementary material.

Supplementary Material 1

## Data Availability

The datasets generated during and/or analyzed during the current study are available from the corresponding author on reasonable request.
