# Supplementary material for: Clinical and radiological effects of Bevacizumab for the treatment of radionecrosis after stereotactic brain radiotherapy
Source: BMC Cancer. 2024 Jul 30;24:918. doi: 10.1186/s12885-024-12643-6 (PMC11290153; doi:10.1186/s12885-024-12643-6)
Supplement: Supplementary file 1 — Supplementary Material 1 [file 12885_2024_12643_MOESM1_ESM.docx]

Supplement Table 1. Effect of analyzed factors on clinical response.

| Factor | No response  Risk ratio and 95% confidence interval (CI) | Significance (p value)  Univariate Multivariate |
| --- | --- | --- |
| ***Age (>55 years)** | **-** | **<0.0001 0.001** |
| Sex (Male) | RR:1.4 95%CI (1-1.9) | 0.061 0.143 |
| Primary tumor (Lung) | RR: 0.4 95%CI (0.1-1.8) | 0.272 - |
| Comorbidity (+) | RR: 1.7 95%CI (1-3.4) | 0.078 0.666 |
| Neurological deficit at RN diagnosis (+) | RR: 1.5 95%CI (0.6-2.3) | 0.057 0.869 |
| BV cycles (<4) | RR:1.4 95%CI (0.3-7.5) | 1 - |
| BV dose (Other vs. 5-7.5 mg/kg) | RR:1 95%CI (0.7-1.4) | 1 - |
| First treatment of RN (Steroid vs. BV) | RR: 2.9 95%CI (0.9-9) | 0.097 0.162 |
| *Timing of BV start (< vs. >2 months) | - | 0.037 0.062 |
| Metastasis diameter (>19.5mm) | RR: 1.6 95%CI (0.6-4.3) | 0.451 - |

*Fisher’s exact test
